# Mediterranean diet adherence in Tunisian psychiatric patients

**DOI:** 10.1192/j.eurpsy.2026.11321

**Published:** 2026-07-31

**Authors:** Y. Ben Othmene, A. Aissa, A. Hlaoui, S. Aouadi, R. Hosni, A. Ouertani, R. Jomli

**Affiliations:** Avicenne psychiatry department, Razi Hospital, Tunis, Tunisia

## Abstract

**Introduction:**

The Mediterranean diet (MedDiet), one of the most studied dietary patterns worldwide, has been associated with a wide range of benefits for physical and mental health.

While its positive impact on the general population is well-documented, there is limited research on adherence to this diet among psychiatric patients and its effects on mental health outcomes.

**Objectives:**

This study aimed to assess adherence to the MedDiet among Tunisian psychiatric patients.

**Methods:**

A cross-sectional descriptive and analytical study, conducted between January and March 2025, involving Tunisian psychiatric patients at the Avicenne psychiatry department of Razi Hospital.

Data were collected using a file and a translated Arabic version of the Mediterranean Diet Adherence Screener (MEDAS). It is a 14-item self-administered questionnaire designed to assess adherence to the MedDiet, categorizing it as: weak (≤5), moderate to fair (6–9), and good to very good (≥10).

**Results:**

Thirty-six male patients were included with a mean age of 37 [19-62] years. Most were single (83%), had not completed secondary education (81%), and were professionally inactive (64%). Smoking was prevalent among 89% of the participants, while 11% reported alcohol consumption.

Only 17% had underlying health conditions, with the most common being hypertension, dyslipidemia and obesity. Psychiatric diagnoses included schizophrenia (50%), bipolar I disorder (39%), and schizoaffective disorder (11%). Regarding treatment, 89% of patients were on atypical antipsychotics, with 25% also receiving typical antipsychotics, 47% were prescribed mood stabilizers and 28% took antidepressants.

In terms of MedDiet adherence, the average MEDAS score was 6.92 (range: 2–10; SD = 2.234). Most patients (67%) exhibited moderate to fair adherence, 22% had weak adherence and 11% showed good to very good adherence.

ANOVA revealed no significant differences in adherence across the psychiatric disorders (p = 0.596) and no significant correlation between adherence and a specific disorder type (p = 0.504).

However, a significant positive correlation was found between age and MedDiet adherence, with older patients showing higher adherence (p = 0.033).

**Conclusions:**

This study found moderate to fair adherence to the MedDiet among Tunisian psychiatric patients, with older patients showing better adherence. These findings highlight the need for further research on the impact of MedDiet on mental health and efforts to improve its adherence, particularly among younger patients.

**Disclosure of Interest:**

None Declared

